# Forefront: MiR-34a-Knockout Mice with Wild Type Hematopoietic Cells, Retain Persistent Fibrosis Following Lung Injury

**DOI:** 10.3390/ijms21062228

**Published:** 2020-03-23

**Authors:** Raanan Bulvik, Moshe Biton, Neville Berkman, Raphael Breuer, Shulamit B. Wallach-Dayan

**Affiliations:** 1 Lung Cellular and Molecular Biology Laboratory, Institute of Pulmonary Medicine, Hadassah—Hebrew University Medical Center, POB 12000, Jerusalem 9112102, Israel; 2 The Lautenberg Center for Immunology and Cancer Research, Institute of Medical Research Israel-Canada, Hadassah Hebrew University Medical Center, Jerusalem 9112102, Israel; 3 Department of Pathology and Laboratory Medicine, 670 Albany St, 4th Floor, Boston University School of Medicine, Boston, MA 02118, USA

**Keywords:** miR34, FLIP, acute lung injury, cell-death, fibroblasts/myofibroblasts, fibrosis resolution, idiopathic pulmonary fibrosis

## Abstract

MicroRNAs (miRs) are known to limit gene expression at the post-transcriptional level and have important roles in the pathogenesis of various conditions, including acute lung injury (ALI) and fibrotic diseases such as idiopathic pulmonary fibrosis (IPF). In this study, we found increased levels of miR-34 at times of fibrosis resolution following injury, in myofibroblasts from Bleomycin-treated mouse lungs, which correlates with susceptibility to cell death induced by immune cells. On the contrary, a substantial downregulation of miR-34 was detected at stages of evolution, when fibroblasts resist cell death. Concomitantly, we found an inverse correlation between miR-34 levels with that of the survival molecule FLICE-like inhibitory protein (FLIP) in lung myofibroblasts from humans with IPF and the experimental model. Forced upregulation of miR-34 with miR-34 mimic in human IPF fibrotic-lung myofibroblasts led to decreased cell survival through downregulation of FLIP. Using chimeric miR-34 knock-out (KO)-C57BL/6 mice with miR34KO myofibroblasts but wild-type (WT) hematopoietic cells, we found, in contrast to WT mice, increased and persistent FLIP levels with a more severe fibrosis and with no signs of resolution as detected in pathology and collagen accumulation. Moreover, a mimic of miR-34a decreased FLIP expression and susceptibility to cell death was regained in miR-34KO fibroblasts. Through this study, we show for the first time an inverse correlation between miR-34a and FLIP expression in myofibroblasts, which affects survival, and accumulation in lung fibrosis. Reprogramming fibrotic-lung myofibroblasts to regain susceptibility to cell-death by specifically increasing their miR34a and downregulating FLIP, may be a useful strategy, enabling tissue regeneration following lung injury.

## 1. Introduction

Pathological fibrosis is associated with increments in molecules such as FLICE-like-inhibitory-protein (FLIP) allowing escape from immune surveillance and unremitted accumulation of myofibroblasts. We have shown, in murine lung myofibroblasts accumulating during evolution of fibrosis, that FLIP mediates the deviation from myofibroblast cell-death and apoptosis towards proliferation [1]. miRNA regulation of FLIP, with subsequent resistance to immune-cell induced cell death has not yet been documented in IPF. miRNAs are a class of evolutionarily conserved non-coding RNAs approximately 20–25 nucleotides in length. It is estimated that up to one third of the human genome may be subject to regulation by miRNAs. Dysregulation of miRNA can contribute to disease pathology [2], including acute lung injury [3,4] and fibrosis [5], in the heart [6,7], kidney [8], liver [9], and lungs [10,11,12,13]. miRNA-34a (miR-34a), specifically, dysregulation has been identified as a modulator of cell-death and apoptosis [14,15]. mirRNA-34a has been linked to diverse types of cancers, and shown to play a role in altered cell-death, uncontrolled proliferation, and metastasis [16,17,18,19,20,21]. 

In this study, using Bleomycin-induced lung injury and fibrosis in chimeric miR34a-knockout mice (miR34a^KO^) with miR34a^KO^ mesenchymal cells and wild type (WT) hematopoietic cells, we determined whether disruption of lung tissue-regeneration following injury, with fibrosis evolution, is associated with downregulation of miR34a levels, specifically in myofibroblasts that upregulate FLIP and resist T-cell induced cell-death. We then set out to assess whether increasing miR34a levels by a specific mimic affects FLIP expression, and cell-death in IPF-lung myofibroblasts. More importantly, we determined that miR34a downregulation links FLIP stability with fibroblast capacity to survive and propagate, and that miR34a mimics may regulate evolution of lung fibrosis following injury. 

## 2. Results

### 2.1. Inverse Correlation between Endogenous MiR-34a and FLIP Levels in Human IPF-Lung Fibroblasts

**Figure 1:**
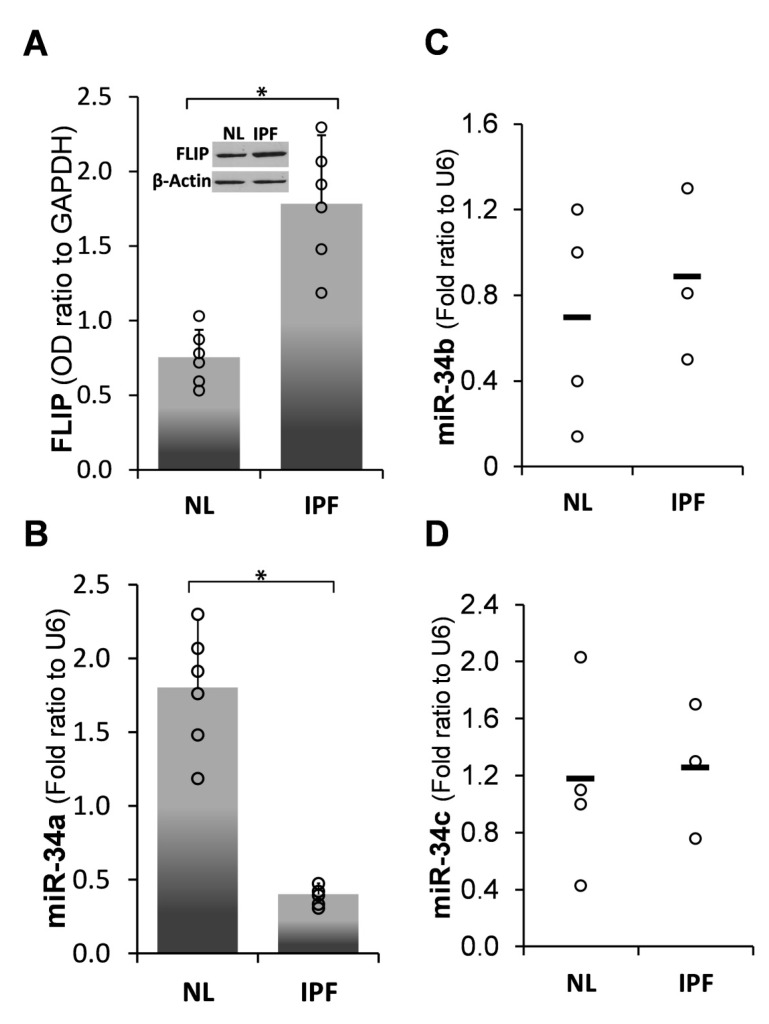
Human idiopathic pulmonary fibrosis (IPF) primary lung-myofibroblasts, compared to normal-, primary-lung myofibroblasts, decrease miR-34a levels and show an inverse correlation with FLICE-like inhibitory protein (FLIP) expression (**A**) Graphical presentation of FLIP protein expression determined by optical densities (OD) in Western blot (WB), and (**B**) qPCR miR-34a expression levels (calculated as 2^−∆∆Ct^) in normal (NL) vs. fibrotic (IPF) lung myofibroblasts. *n* = 5–6. (**C**) qPCR for miR-34b and (**D**) miR-34c in NL and IPF human lung myofibroblasts. Error bars represent mean ± SD; circles represent individual data points. * *p* < 0.01.

miR-34a is a known regulator of cell-death and apoptosis [14,15]. FLIP expression increased in human IPF- compared to normal-lung myofibroblasts, from 0.8 ± 0.15 SD (*n* = 5) to 1.7 ± 0.5 SD (*n* = 6), respectively (Figure 1A, NL vs. IPF). In contrast, we detected a substantial decrease in miR-34a levels from 1.8 ± 1 SD to only 0.4 ± 0.2 SD (*n* = 3), in human IPF- compared to normal-lung myofibroblasts, respectively (Figure 1B, NL vs. IPF), (Pearson coefficient of inverse correlation [R] = −0.6). Of note, no difference in the levels of miR-34B or miR-34C were detected between these cells (Figure 1C,D, respectively). 

### 2.2. Forced miR-34a Overexpression, Using a miR34a Mimic, Mediates FLIP Downregulation in IPF-lung Myofibroblasts

**Figure 2:**
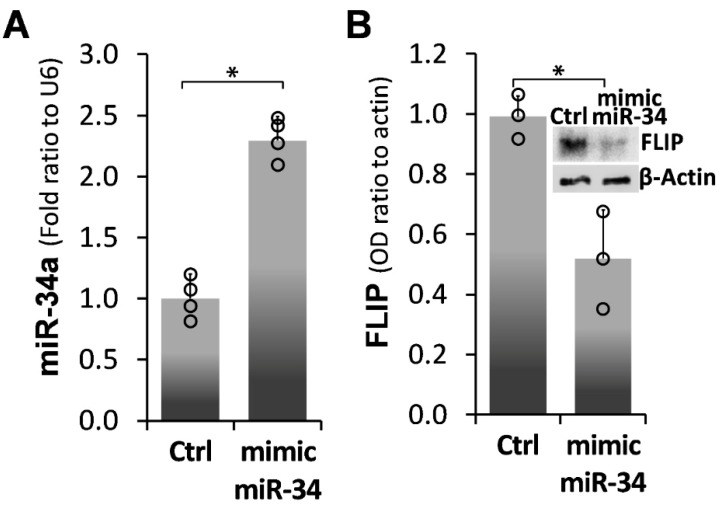
miR-34a mimic-forced miR-34a overexpression in fibrotic IPF lung myofibroblasts decreases FLIP levels. (**A**) Graphical presentation of miR-34a levels in qPCR (calculated as 2^−∆∆Ct^) in mimic miR-34a-transfected CCL-191 cells (IPF cell line). (**B**) Graphical presentation and Western blot (**B;** insert) of FLIP expression. Representative results from four independent experiments. Error bars represent mean ± SD; circles represent individual data points. * *p* < 0.01–0.05.

miR-34a over-expression was induced, in IPF fibrotic-lung myofibroblast cell line (i.e., CCL-191), following transfection with a mimic miR-34a. qPCR results show a substantial, ~2.5-fold increase in miR-34a levels, from 1 ± 0.2 SD to 2.3 ± 0.2 SD (*p* < 0.05), in miR-34a transfected cells (mimic miR-34a) compared to those transfected with a negative control (Ctrl) (Figure 2A). Fibrotic-lung fibroblasts transfected with mimic-miR34a substantially reduced FLIP protein levels in Western blot (Wb) by 50%, from 1 ± 0.08 SD to 0.5 ± 0.2 SD (*p* < 0.05) (Figure 2B; graph and insert of FLIP Wb in mimic miR-34 vs. Ctrl). These results establish that miR-34a plays a role in the regulation of FLIP protein expression.

### 2.3. Mimic MiR34a Increases Cell Death in IPF-Lung Myofibroblasts

**Figure 3:**
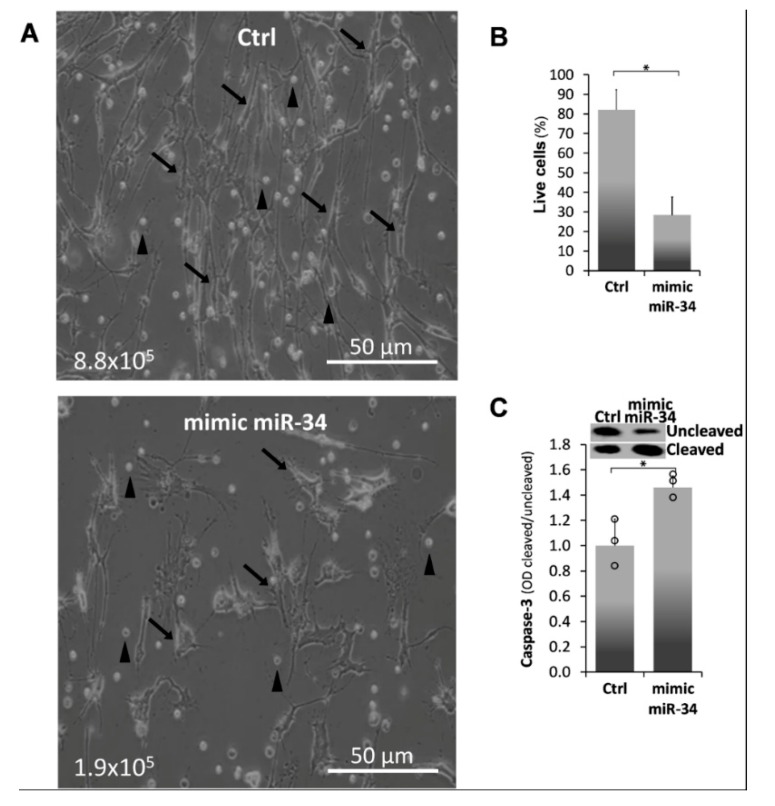
Mimic miR-34a increases cell-death in human IPF-lung fibroblasts Work flow; fibrotic-lung myofibroblasts (ATCC-CCL-191) were transfected with mimic miR34a (5 nM, 72h, Trans-iT) vs. control (NC) and then co-cultured with Jurkat T cells. (**A**) Microscope images with trypan-blue exclusion (arrows denote myofibroblasts; triangles, lymphocytes) (**B**), graphical presentation of cell survival percentage and (**C**), Graphical presentation of cleaved/uncleaved caspase-3 ODs (Upper panel) and Wb (insert). Representative results from three independent experiments. Error bars represent mean ± SD; circles represent individual data points. * *p* < 0.01–0.05.

We assessed cell death in mimic miR-34a-transfected CCL-191 IPF-lung myofibroblasts, with decreased FLIP (Figure 2), following co-culture with Jurkat T-cells (1:2, respectively). Following mimic miR-34a-transfection, cell counts detected by trypan blue exclusion, decreased from 8.8 × 10^5^ to only 1.9 × 10^5^, compared to negative control-treated cells (Ctrl) (Figure 3A, Ctrl vs. mimic miR34, respectively). The calculated cell survival decreased from 80% to only 25% (Figure 3B, Ctrl vs. mimic miR-34, respectively). 

Cell-death was further verified by caspase-3 cleavage in Wb, and graphically summarized. The ratio between cleaved/uncleaved caspase3 was increased in myofibroblasts transfected with miR-34a from 1 ± 0.2 SD to 1.5 ± 0.1SD (*p* < 0.05) compared to controls (Figure 3C, Ctrl vs. mimic miR-34). Thus, human fibrotic lung myofibroblasts that overexpress miR-34a are more susceptible to T-cell induced cell-death.

### 2.4. Kinetic Profiling of MiR-34a Following Exposure of C57BL/6 WT Mice to Bleomycin, Reveals an Inverse Correlation with FLIP Expression Which Is Reduced at Times of Evolution of Fibrosis and Surges to Normal Levels at Times of Resolution

**Figure 4:**
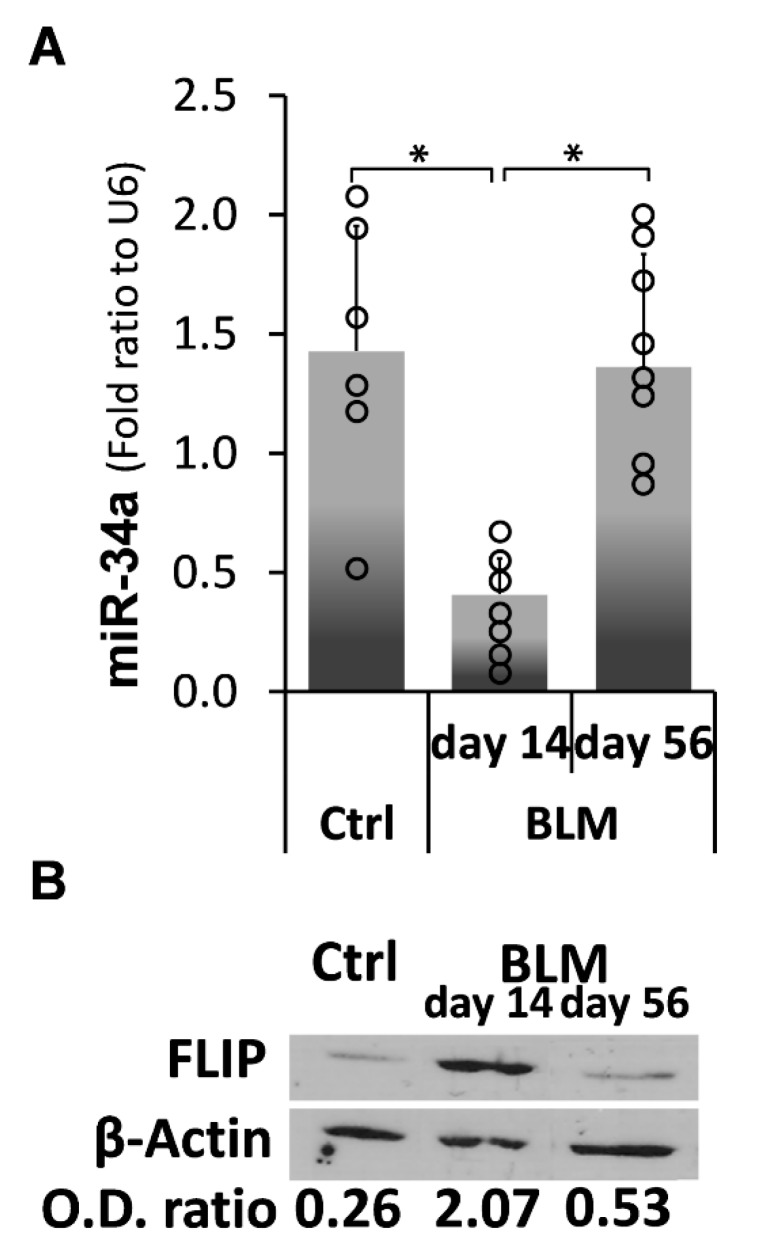
Kinetics profile of miR-34a, in Bleomycin-treated mice shows reduction at times of fibrosis evolution and upsurge to normal levels at resolution, and an inverse correlation with FLIP. C57BL/6 wild-type mice were exposed to Bleomycin (0.03 mU/mice) or control-saline, sacrificed 14, 56 days post Bleomycin-OA, and myofibroblasts were isolated. (**A**) Total RNA was extracted and qPCR miR-34a expression levels determined (calculated as 2^−∆∆Ct^). (**B**) Myofibroblast cell lysates were obtained to assess FLIP in Wb using anti-FLIP mAb. OD ratios, normalized to β-actin at each time point, are presented. One representative of four experiments with similar results; *n* = 5–6 for each time point; error bars represent mean ± SD; circles represent individual data points. * *p* < 0.001.

We then determined the kinetics of miR-34a vs. FLIP expression in lung myofibroblasts in the experimental model Bleomycin-induced lung injury compared to control, saline-treated, C57BL/6 mice. Lung myofibroblasts were isolated from Bleomycin (BLM)-injured lungs, at times when fibrosis was evolving (14 days post BLM) and resolving (56 days post BLM). qPCR quantification for miR-34a revealed, as in myofibroblasts from lungs of humans with IPF vs. normal lungs (Figure 1), that control saline-treated mice had relatively high miR-34a levels, which decreased from 1.45 ± 0.8 SD to only 0.3 ± 0.1 SD (*n* = 6), on day 14 (Figure 4A, BLM d-14 vs. Ctrl). Moreover, and in contrast to lungs from IPF that do not resolve fibrosis, on day-56 when fibrosis resolves in the experimental model, miR-34a increased to 1.35 ± 0.9 SD and returned to normal levels (Figure 4A, BLM d14 vs. d56 and control saline-Ctrl). In contrast, we detected a substantial increase in FLIP levels from an OD of 0.26 to 2.07 (*n* = 12) on day 14 (Figure 4B, BLM d-14 vs. Ctrl) to an OD of 0.53, the normal level detected on control saline (Figure 4B, BLM d-56 vs. d-14 and Ctrl). These results show an inverse correlation between myofibroblast miR-34a and FLIP levels during evolution as well as during resolution of fibrosis in the experimental model of Bleomycin-induced lung injury. Moreover, on day 56, when fibrosis resolves, miR-34a and FLIP return to normal levels, meaning that miR-34a level is high, and FLIP is low.

### 2.5. Chimeric MiR-34a Knockout Mice (with Mesenchymal Cells Lacking MiR-34a), Compared to Control WT Mice, Express Consistently High FLIP Levels and Are More Sensitive to Bleomycin-Induced Lung Injury, and Do Not Resolve Fibrosis

Our next goal was to evaluate Bleomycin-induced lung injury and fibrosis evolution/resolution in the absence of miR-34a, specifically in mesenchymal cells (e.g., myofibroblasts). To this end, we generated a chimeric miR-34^KO^ mouse with WT immune cells and miR-34^KO^ mesenchymal cells (see methods). Chimerism efficiency has been proven as we previously detailed (data not shown) using GFP^+^ WT mice [22]. Bleomycin was introduced into both the chimeric miR-34^KO^ and WT groups, and lung regeneration vs. fibrosis severity was evaluated at times of fibrosis evolution (day 14) and resolution (day 56). 

**Figure 5:**
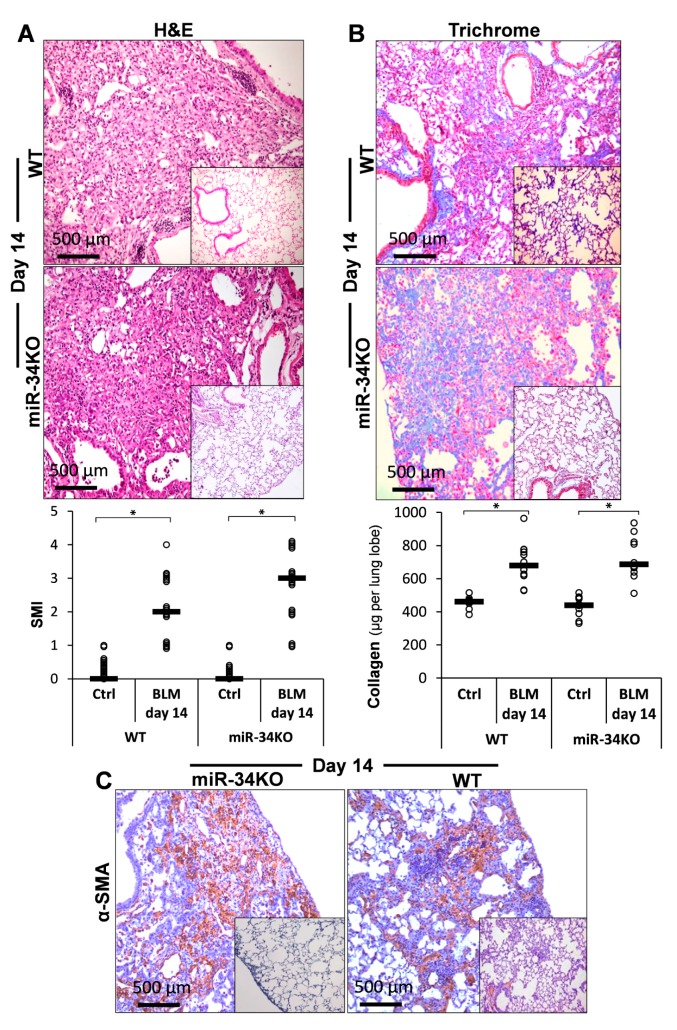
Chimeric miR-34aKO mice, compared to wild type (WT) are slightly more sensitive to Bleomycin-induced fibrosis. (**A**) Hematoxylin and eosin (H&E) staining at day 14 post-BLM vs. saline (SAL) (Upper panel), with graphical presentation of a semi-morphological index (SMI) (Lower panel), in WT and miR-34^KO^ murine lung tissue sections. (**B**) Masson-trichrome (Upper panel, blue) and (**C**) αSMA (brown) IHC and (**B;** Lower panel), graphical presentation of Sircoll-collagen assay. Bars (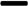) represent median score. Representative results from three independent experiments. Error bars represent mean ± SD; circles represent individual data points. * *p* ≤ 0.01–0.05.

Chimeric miR-34^KO^ mice had pronounced lung fibrosis approximately similar to the WT, in hematoxylin and eosin (H&E)-stained sections (Figure 5A, upper panel, WT vs. miR-34^KO^ vs. saline inserts), with the mean SMI grade increasing from two to three, on average (Figure 5A, lower panel, graphical presentation, WT and miR-34^KO^ vs. Ctrl). Collagen increments with Masson-trichrome staining of lung tissue sections (Figure 5B, upper panel, Trichrome, WT vs. miR-34^KO^ vs. saline inserts), or using Sircol-red assay of soluble collagen (Figure 5B, lower panel, graphical presentation, WT and miR-34^KO^, BLM vs. Ctrl), were similar in lungs of WT- and miR-34^KO^- mice. α-SMA staining in lung tissue sections was also almost similar for the two groups of mice (Figure 5C, α-SMA, WT vs. miR-34^KO^ vs. saline-inserts). These findings suggest that poor miR-34 levels result in a slightly more pronounced fibrosis.

**Figure 6:**
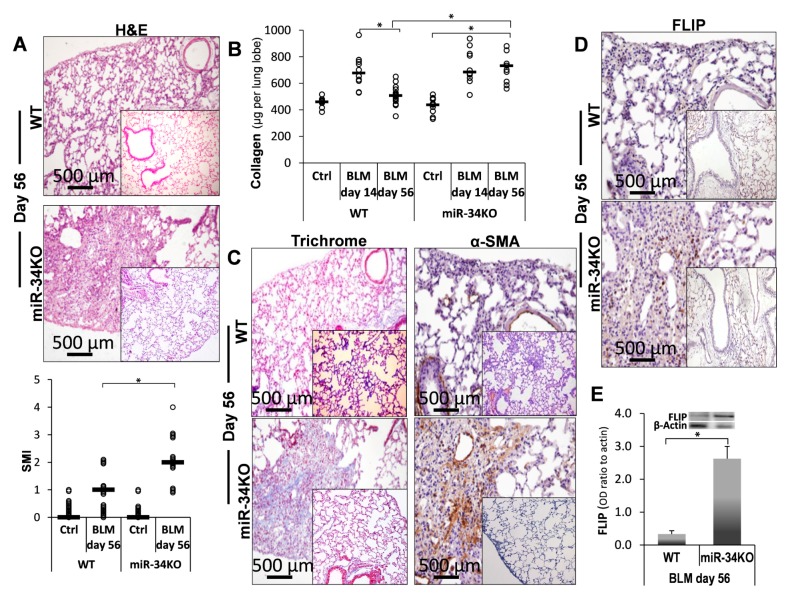
Persistent fibrosis in Bleomycin-treated miR-34^KO^ as opposed to recovery in WT mice. (**A**) H&E staining (Upper panels), with graphical presentation of SMI-H&E (Lower panel), at day 56 post-BLM (vs. saline, inserts), in WT and miR-34^KO^ murine lung tissue sections. (**B**) Sircoll-collagen assay at indicated days, and (**C**) Masson-Trichrome (blue), with αSMA (brown). (**D**) FLIP (brown) IHC at day 56 post-BLM in WT, and miR-34^KO^ mice lung tissue sections, with (**E**) Western blot graphical presentation of FLIP levels in WT and miR-34^KO^ in isolated lung fibroblasts on day-56. *n* = 3–4 for each time point. Representative results from three independent experiments. Error bars represent mean ± SD; circles represent individual data points. * *p* ≤ 0.01–0.05.

However, at 56 days post Bleomycin, compared to control saline (Ctrl), we detected persistent fibrosis with no sign of resolution in chimeric miR-34a^KO^ mice, in contrast to WT mice which had recovered from fibrosis at this time point (Figure 6, miR-34^KO^ vs. WT). In particular, the Bleomycin-treated chimeric miR34^KO^ mice had advanced fibrosis with severe histopathology detected in H&E staining (Figure 6A upper panels, miR-34^KO^ vs. WT vs. saline-inserts) and an average SMI score of 2.2 in chimeric miR-34^KO^ vs. an SMI of 1 in the control WT mice (Figure 6A, lower panel, graphical presentation, WT and miR-34^KO^ vs. Ctrl). Likewise, in the WT, collagen levels on day 56 had returned to the levels detected in normal saline-treated mice (Figure 6B, upper panel, WT-day56 vs. WT-day14 and WT-Ctrl); however, there was comparable and persistent lung collagen deposition in the lungs of chimeric miR34a^KO^ mice at day 56 compared to day 14, with acute fibrosis (Figure 6B, upper panel, miR-34^KO^ -day56 vs. miR-34^KO^ -day14 and WT-Ctrl). In addition, increased trichrome and α-SMA staining were detected on day 56 in chimeric miR-34^KO^ mice (Figure 6C, miR-34^KO^ vs. WT vs. Ctrl-inserts). Moreover, at this time point, in contrast to day 14 where we have shown increased FLIP levels specifically in fibroblasts using counterstaining of α-SMA cells and FLIP [1], WT mice had significantly lower FLIP levels than chimeric miR-34^KO^ mice, as assessed by IHC in lung tissue sections (Figure 6D, miR-34^KO^ vs. WT) and in Western blot, in isolated lung myofibroblasts, with an OD-ratio to *β*-actin of 0.33 and 2.5, respectively (Figure 6E, miR-34^KO^ vs. WT). These results led us to conclude that chimeric miR-34^KO^ mice lungs do not regenerate or resolve fibrosis, unlike WT mice, possibly due to persistent upregulation of FLIP expression. Moreover, as IPF lungs, which show low levels of miR-34a, bleomycin-treated miR-34a knockout mice with even lower miR-34a levels increase SMI and collagen, indicating severe fibrosis in the absence of miR-34a.

### 2.6. Mimic-MiR34a, Decreases FLIP, and Restores Sensitivity to Cell-Death in MiR34aKO Murine Lung Myofibroblasts

**Figure 7:**
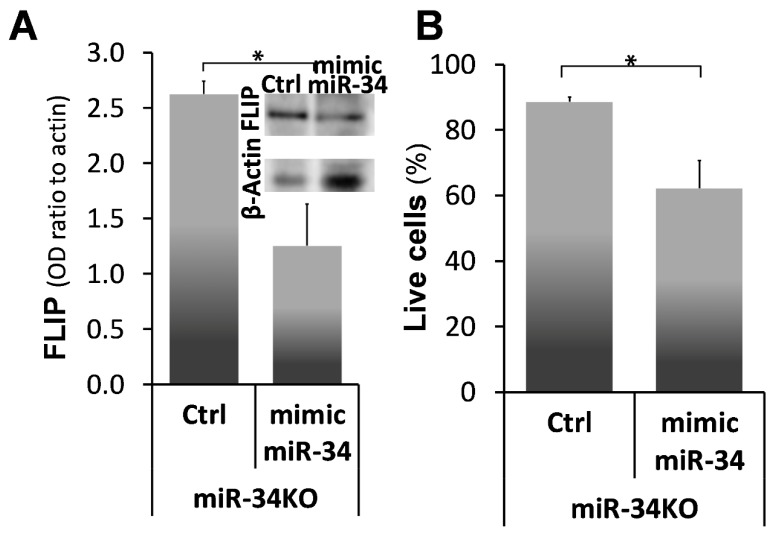
miR-34a mimic-mediated miR-34a overexpression and FLIP downregulation with decreased survival, in miR-34^KO^ myofibroblasts. (**A**) Graphical presentation of FLIP expression normalized to β-actin and Western blot (A; insert). One representative of four experiments with similar results. (**B**) Trypan-blue exclusion following co-culture with Jurkat T-cells for mimic miR34a (5 nM, 72 h, Trans-iT)—(mimic miR-34) vs. control (Ctrl) transfected myofibroblasts from miR-34^KO^ mice (miR-34KO), at day 56 post Bleomycin. Representative results from two independent experiments. Error bars represent mean ± SD; * *p* < 0.001.

Next, FLIP levels and susceptibility to lymphocyte-induced cell death were assessed after mimic miR34a transfection in myofibroblasts isolated from lungs of the chimeric miR-34^KO^ Bleomycin-treated mice (Figure 7). Comparisons between miR34aKO lung myofibroblasts treated with mimic miR-34 vs. controls were made at day 56, a time point when chimeric miR34aKO mice did not resolve fibrosis compared to the WT mice (see Figure 6). miR-34^KO^ myofibroblasts, treated with negative control (Ctrl), show high FLIP levels 2.25 times higher than those treated with miR-34 mimic (Figure 7A; miR-34-KO + Ctrl vs. miR-34-KO + mimic miR-34, graph and insert). These results were identical to those in their WT myofibroblast counterparts (see Figure 4). Cell survival was assessed also in this experiment by trypan blue exclusion following co-culture with Jurkat T-cells (1:2). Myofibroblasts isolated on day 56 from miR-34^KO^ mice, that were transfected with mimic miR-34a, had shorter survival rates of 60% ± 6 SD, compared to 89% ± 0.5SD (Figure 7B; miR-34KO + Ctrl vs. miR-34KO +mimic miR-34). Thus, overexpression of miR-34a resulted in downregulation of FLIP, which allows myofibroblasts to regain the ability to undergo cell death in co-culture with T-cells.

## 3. Discussion

It is known that acute lung injury is a pathology that may serve as a trigger for a number of pulmonary fibrotic conditions, such as pulmonary emphysema, chronic obstructive pulmonary disease, interstitial pneumonia, and idiopathic pulmonary fibrosis [23,24,25,26]; on the other hand, acute exacerbations are one of the complications of pulmonary fibrosis [27,28,29]. Pathological fibrosis is associated with increments in molecules such as FLIP, allowing unremitted accumulation of myofibroblasts. We formerly validated, in myofibroblasts accumulating during evolution of lung fibrosis, that FLIP mediates the deviation of cell death to proliferation and thus facilitates escape from immune surveillance [1]. In this study, we further determined the role of miR-34a on FLIP expression in human IPF lung myofibroblasts, in vitro, and in mouse lung myofibroblasts in vitro and in vivo, following Bleomycin-induced lung injury in chimeric-miR-34aKO mice, with miR-34aKO specifically in mesenchymal cells, compared to their WT counterparts. We found an inverse correlation between miR-34a levels and FLIP, in myofibroblasts isolated from lungs of humans with IPF or from mice during the evolution and resolution phases of fibrosis. Moreover, lung myofibroblasts from patients with IPF, which expressed high FLIP levels while miR-34a levels were low (Figure 1), and those treated with mimic miR-34a, downregulated FLIP (Figure 2), exhibited increased susceptibility to T-cell mediated cell death (Figure 3). In WT mice, lung fibrosis evolved at days 7–14 after Bleomycin exposure, when miR-34a is downregulated, but resolved at days 28 to 56, when miR-34a returned to pre-injury levels (Figure 4). 

Unlike myofibroblasts from WT mice, those from chimeric miR-34aKO mice persistently expressed high FLIP levels, from day 14 to 56. They were also slightly more sensitive to, and more importantly, they did not resolve fibrosis, following Bleomycin-induced lung injury (Figure 5 and Figure 6). Concomitantly, in a mirror experiment, following miR-34a upregulation with a specific mimic, we show that there is an inverse correlation between the levels of miR-34a and FLIP and that miR-34a increases susceptibility to Jurkat T-cell induced cell death in myofibroblasts from lungs of humans with IPF as well as of miR34aKO mice (Figure 7). This may imply direct or indirect downregulation of FLIP by miR-34a and requires further investigation. It needs to be mentioned, however, that the model in this study was miR-34a whole-body knockout mouse with wild-type hematopoietic cells, which means that mesenchymal cells, epithelial cells, endothelial cells, etc., were also miR-34a knockout. Hence, the results observed in vivo could reflect miR-34-knockout effects on a variety of mesenchymal cells and others. 

Interestingly, a recent study found that miR-34a is upregulated in alveolar epithelial cells, but not in lung fibroblasts, of aged mice treated with Bleomycin [30,31]. Epithelial–mesenchymal transition (EMT) is defined as a developmental process that shifts cellular differentiation from an epithelial to a mesenchymal cell type [32]. EMT was found to contribute to IPF pathology by promoting increased secretion of extracellular matrix components by alveolar epithelial cells, thereby directly influencing the local fibrotic process [33]. Although our work did not focus on EMT, the contribution of miR-34a to this process was studied by Rokavec et al. [34]. Moreover, in a previous study by Cha et al. [35] a compartmentalized expression of c-FLIP was observed, with increased expression in alveolar epithelial cells surrounding fibrotic foci and diminished expression in myofibroblasts. In this study and based on our previous results [1] we concluded that fibroblasts resistance to cell death was dependent of c-FLIP expression. These contradictory results probably imply a cell-specific phenomenon with versatile phenotypes on a cell-specific basis and it may be that FLIP allows EMT by protecting epithelial cells from cell death during transdifferention into mesenchymal cells (e.g., fibroblasts). Thus, miR-34a might be playing a role in other stromal cells (especially the epithelium). Cui et al. showed that repression of miR-34a was necessary for EMT, invasion, and metastasis in colorectal cancer. In addition, while there were some differences in human IPF lung myofibroblasts including differences in the timing and kinetics of the evolution and resolution of fibrosis and miR34a expression, Cui et al. [30] showed that myofibroblast cell death was diminished and fibrosis persisted in the lungs of Bleomycin-treated miR34KO mice. Their findings are consistent with our observations in this study in chimeric-miR34KO mice. While we refer to day 14 as the time point when fibrosis is at its peak, Cui’s group refer to day 21 as the peak of the disease. At that time point, we start to see resolution of fibrosis. In our study, miR-34a levels were lower in the fibroblast of IPF patients (Figure 1). The differences in miR-34a levels between literature and our results could be explained by differences between expression phenotypes between various disease profiles of the patients sampled for the respective studies [36]. In their study, Cui et al. found that miR-34a demonstrated greater expression in the lungs of patients with IPF and in mice with experimental pulmonary fibrosis, with its primary localization in lung fibroblasts. More importantly, miR-34a was up-regulated significantly in both human and mouse lung myofibroblasts. Nevertheless,, in another study, which supports our results in this article, they found that upregulation of miR-34 is important for alleviation of fibrosis and acts in a negative feedback mechanism to restrain pulmonary fibrosis in a Bleomycin-induced mouse model [37].

miRs have been associated with many basic cellular processes such as cell death, proliferation, and differentiation [6,38,39]. Some miRNA-based therapeutics have already entered Phase 2 clinical trials, such as miR-122 antagonists in hepatitis C virus [18,40,41], holding the promise of a new class of therapeutic drugs. Wang et al. summarized the contradictory role of miR-34 and its adverse roles in vascular diseases [42]. Moreover, several studies have implicated microRNAs in lung fibrosis, including mir29, let-7d, mir17-92. These findings were proposed to be translated into clinics with a potential blocking for pulmonary fibrosis [43].

In our context, transfection of human fibrotic lung myofibroblasts with mimic miR-34a resulted in decreased FLIP levels (Figure 7), indicating that mimics of miR-34a in myofibroblasts in in injured lungs or with fibrosis, may pave the way to tackling myofibroblast resistance to cell death and subsequent fibrosis. 

## 4. Materials and Methods

### 4.1. Human Subjects and Lung Biopsies 

Primary cultures of human fibroblasts derived from lungs of IPF patients and healthy individuals were purchased from Carol Feghali-Bostwick (Medical University of South Carolina, Charleston, SC, USA). Informed consent was obtained under a protocol approved by the Institutional Review Board for Human Research at the Medical University of South Carolina. All patients fulfilled the criteria for the diagnosis of IPF as established by the American Thoracic Society and the European Respiratory Society [44]. Control specimens were collected during surgical resection of solitary pulmonary nodules, and lung cancer was excluded by pathology after surgery [45]. 

Cells from the IPF lung myofibroblast cell line CCL-191, the normal lung myofibroblast cell line CCL-151, and the human Jurkat T cell line were procured from American Type Culture Collection (ATCC, Manassas, VA, USA). 

Human and mouse lung myofibroblasts and Jurkat cells were cultured in RPMI 1640 fibroblast culture medium (Merck Group, Darmstadt, Germany), and were incubated at 37 °C in 5% humidified CO_2_. Cells were passaged two times per week by dissociating monolayers with a mild trypsin solution and were used between passages three and six.

### 4.2. Animals

Male or female 11–12-week-old C57BL/6J WT mice, (Harlan Ltd., Jerusalem, Israel), and male or female 11–12-week-old miR-34a dominant negative mice with deleted miR-34a pre-miRNA expression were used. (miR-34a dominant negative mice were kindly provided by Prof. Yinon Ben-Neria, Hebrew University, Jerusalem, Israel. For background information see https://www.nature.com/articles/ncb2366#methods.) Mice were maintained under specific pathogen-free conditions in the Animal Unit of the Hebrew University-Hadassah School of Medicine with adherence to institutional guidelines for the care and use of laboratory animals. The experimental work was performed with approval of the Hebrew University-Hadassah Institutional Animal Care and Use Committee (ethics approval No. MD-15-14590-5, granted on 16 December 2015). Chimeric mice were generated as described previously by our lab [22]. Briefly, C57BL/6J WT mice or miR-34^KO^ mice were subjected to sublethal total body irradiation (750 cGy) delivered by a linear accelerator (G6, dose rate 179 cGy/min, source-to-skin distance 80 cm) (Clinac, Varian, Palo Alto, CA, USA). The mice received intravenous instillation of syngeneic splenocytes (10–20 × 10^6^) obtained from WT donor mice 1 day after irradiation, creating chimeric miR-34^KO^ mice (miR-34^KO^ in all cells except for spleen maturating hematopoietic cells, which are of WT origin). For controls, the same procedure was performed with WT mice. Mice were treated with Bleomycin to induce fibrosis 1 month after chimeras were established.

### 4.3. Oropharyngeal Aspiration and Induction of Lung Fibrosis in Mice

We performed oropharyngeal aspiration of Bleomycin (OA-BLM) as previously detailed [46,47]. Briefly, animals were lightly anesthetized with 2% isoflurane delivered in a box. Mice were then fixed on a surgery board, the tongue was pulled out with forceps, and 0.1 mL of BLM (0.03 mU) was placed onto the distal part of the oropharynx while the nose was gently closed. Mice were sacrificed by a lethal dose of pentothal (CTS—Kiryat Malachi, Israel) (100 mg per mouse in 0.5 mL 0.9% saline) at different time points following OA-BLM. After sacrifice, left lungs provided tissue sections for pathological examination (“lung injury”), bronchoalveolar lavage (BAL), and assessment of myofibroblast accumulation. Lower right lobes were used for fibroblast isolation and upper right lobes for collagen assays. Lung injury was assessed quantitatively, as previously described by our laboratory in BAL studies, using a semi-quantitative morphological index (SMI) and quantitative morphological studies of lung fibrosis [48,49]. Lung collagen was measured using a Sircol Collagen Assay kit (Cat. No. S1000, Biocolor, Belfast, Northern Ireland), as described previously [50,51].

### 4.4. Isolation of Lung Myofibroblasts

Mouse myofibroblast isolation has been described by us in detail elsewhere [22,49]. Briefly, lung tissue was minced and incubated with PBS-buffered collagenase solution to release cells. The lung cells were resuspended in RPMI 1640 fibroblast growth medium supplemented with 10% FCS and allowed to attach. 

### 4.5. Lymphocyte–Myofibroblast Coculture Experiments

Mouse lung myofibroblasts and CD4^+^ T cells were isolated ex vivo as described above. Myofibroblasts were plated (4–6 × 10^5^) on six-well tissue culture plates coated with 0.2% gelatin in 2 mL of 2% FCS RPMI medium. Lymphocytes were added when the myofibroblasts reached 70–85% confluence, as described elsewhere [22,49], with or without the addition of 10 μM anti-mouse MFL-3 or anti-human NOK-1 antagonist to FasL mAb. Following 24–48 h of coculture, the supernatant, which contained dead myofibroblasts as well as lymphocytes, was discarded. Adhered myofibroblasts were analyzed to detect initiation of apoptosis by Annexin V staining and flow cytometry and for cell survival and cell death, by trypan blue exclusion. 

### 4.6. In Vitro Detection of Apoptotic and Dead Myofibroblasts

Trypan blue exclusion—Cells were exposed to trypan blue (0.04% in 1 × PBS) and examined using a hemocytometer under a light microscopy. The percentage of surviving myofibroblasts was determined by counting the percentage of unstained cells. 

Caspase-3 cleavage in Western blot—Cells were collected for lysis on ice in RIPA buffer. After centrifugation at 12,000× *g* for 15 min, the supernatant was collected and the protein concentration was determined by BCA protein assay kit (Cat. No. P0012, Beyotime, Haimen, Jiangsu, China). An equal amount of total protein was separated on an SDS-polyacrylamide gel and transferred to a polyvinylidene difluoride (PVDF) membrane (Bio-Rad, Hercules, CA, USA). The membrane was blocked with blocking buffer (5% nonfat milk powder in Tris-buffered saline/Tween 20) for 1 h at room temperature, and then incubated with primary caspase 3, which also detects the cleaved caspase3 (1:1000, Cell Signaling, Beverly, MA, USA).

### 4.7. Immunohistochemical Staining of Lung Tissue Sections

Lung section immunohistochemistry techniques were as previously described [22,49]. Briefly, the slides were incubated with 5% H_2_O_2_ solution to block endogenous peroxidase. The antigen retrieval was performed in 10 mMol/L citrate buffer, after which the slides were incubated with the blocking reagent A of the N-Histofine kit (Cat. No. 414341F, Nichirei Bioscience, Tokyo, Japan). The slides were then incubated with the corresponding antibody solution (anti-α-SMA (Cat. No. M0851, DAKO Agilent, Santa Clara, CA, USA) and anti-FLIP (Cat. No. AF821, R&D Systems, Minneapolis, MN, USA)) in PBS-buffered 1% BSA solution, blocked in the blocking reagent B, and incubated with the secondary antibody. The staining was performed with DAB solution, and the counterstaining with hematoxylin.

### 4.8. Ariol Imaging

Immunohistochemistry slides stained with αSMA mAb were digitized using a commercial image analysis system (Ariol, Genetix, New Milton, UK) and SMI was assessed using an automated system (Ariol), as described elsewhere [52]. Specimens were scanned at low- (1.25×) and high-resolution (20×), using a BX 61 microscope (Olympus, Tokyo, Japan) with an automated platform (Prior Scientific, Rockland, MA, USA). Slide loading was automated (SL50, Genetix/Molecular Devices, Sunnyvale, CA, USA). The system was programmed to select stained and unstained cells and nuclei by color and nuclear shape in high-resolution images. Brown staining was considered positive and blue staining was considered negative. αSMA cell-staining intensity and percentage were measured. SMI scores reflect area, brown staining reflects intensity. 

### 4.9. Assessment of FLIP Protein Levels in Lung Myofibroblasts

FLIP was determined in myofibroblasts isolated from lungs of humans with IPF and from mice at different time points after OA-BLM by Western blot, as we described previously [53] using anti-FLIP antibody (Cat. No. AF821, R&D Systems, Minneapolis, MN, USA). 

### 4.10. Cell lysis and Protein Immunoblotting

Cells were lysed in NP-40 buffer containing 50 mmol/L Tris pH 7.5, 150 mmol/L NaCl, 0.3% 4-nonylphenyl-polyethylene glycol (Nonidet, P-40), 1 μg/mL aprotinin, 1 μg/mL leupeptin, 1 μg/mL pepstatin, 1 mmol/L Na_3_VO_4_, and 1 mmol/L phenylmethylsulfonylfluoride (PMSF). Cell lysates were subjected to sodium dodecyl sulfate polyacrylamide gel electrophoresis (SDS-PAGE). Western blotting (protein immunoblotting) was performed according to the standard protocol.

### 4.11. Mimic–MiR34a Transfection

Transfection by mimic-miR34a was carried out using miRIDIAN microRNA hsa-miR-34a-3p mimic (Cat. No. CS-002040-01, Dharmacon, Tamar, Israel) bearing the following sequence: 5’-UGGCAGUGUC UUAGCUGGUUGU-3’. This mimic is labeled with fluorescein on the 3’-antisense strand for detection of positive transfection. This sequence is conserved among species; therefore, it was also used for mouse samples. For control miRIDIAN miRNA, mimic negative control-1 (Cat. No. CS-002040-01, Dharmacon, Tamar, Israel) was used.

Following the cell-seeding day, cells were transfected either with mimic miR-34a or negative control sequence to a final concentration of 50 nM using Trans-IT-X2 transfection reagent (MIR 6003, Mirus, Zotal, Israel) according to the manufacturer’s recommendations. 

### 4.12. Real-Time PCR of MiRNAs

miRNEASY kit (Qiagen, Hilden, Germany) was used for isolation of total RNA and mature miRNA from myofibroblasts, which was set by a two-step RT-PCR as published previously [54]. Briefly, addition of poly-adenosine to total-RNA (500 μg) was performed using the poly(A) polymerase (Poly(A) Tailing Kit (New England Biolabs, Ipswich, MA, USA) according to manufacturer’s directions. Reverse-transcription was performed using 0.5 μg poly (T)-adapter (5’-GCGAGCACAGAA TTAATACGACTCACTATAGG(T)12VN-3), resulting in cDNA ready for RT-PCR using the miR-34a specific forward primer and the sequence complementary to the poly(T)-adapter as the reverse primer. U6 snRNA were used as the endogenous reference genes for PCR quantification. PCR conditions were as follows: an initial denaturation step at 95 °C for 10 min; 40 cycles of denaturation at 94 °C for 15 s, hybridization at 58 °C for 25 s, and elongation at 72 °C for 25 s. A melting curve was used for the dissociation stage. Gene expression was calculated using delta-delta CT (2^–∆∆Ct^) method.

### 4.13. RNA Analysis and Quantitative PCR (qPCR)

Total RNA was extracted from fibroblasts by RNeasy Kit (Cat. No. 74104, Qiagen, Hilden, Germany), and subjected to reverse transcription using M-MLV-RT (Quanta, Biological Industries, Beit HaEmek, Israel). Quantitative mRNA expression levels were analyzed using real-time PCR (Rotergene, Qiagen), with SYBR GREEN (Cat. No. A46012, Agentec, Applied Biosystems, Warrington, UK). RT-PCR primers were designed to an exon-exon boundary in all indicated transcripts when possible (Table 1).

### 4.14. Data Analysis and Statistics

The nonparametric Kruskall-Wallis test was applied to compare variables measured at different time intervals or following different treatments. Multiple pairwise comparisons were performed using the Mann–Whitney nonparametric test with the Bonferroni correction for significance. The Spearman nonparametric correlation coefficient was calculated to assess associations between pairs of variables. The results were graphically presented in rank values. Two-way ANOVA was used to assess time and treatment effects, and the interaction between them. Using this statistical model, the Scheffe post-hoc procedure was applied for multiple pairwise comparisons. 

All statistical tests were two-tailed, and a *p*-value of 0.05 or less was considered significant.

## 5. Conclusions

Our work shows for the first time that miR-34a affects FLIP protein levels. Our results indicate that during IPF this repression of miR-34a leads to myofibroblast resistance to T-cell initiated cell death and accumulation, resulting in fibrosis. Recovery of miR-34a levels, specifically on fibroblasts, would result in low FLIP levels, suppressing their resistance to cell death and their escape from immune surveillance allowing fibrosis resolution.

## Figures and Tables

**Table 1:** Primers for qPCR.

Gene Species	Primer: Forward	Primer: Reverse
Murine/human U6 snRNA	GACTATCATATGCTTACCGT	GCGAGCACAGAATTAATACGAC
Murine/human miR-34a	TTGCAGTGTCTTAGC TGGTTGTT	CGAGCACAGAATTAATACGAC
Human HPRT	TGACACTGGCAAAACAATGCA	GGTCCTTTTCACCAGCAAGCT
Murine HPRT	GTTAAGCAGTACAGCCCCAAA	GGGCATATCCAACAACAAACTT
